# Relative Validity of the Meal-Based Diet History Questionnaire for Estimating Nutrient Intake among Japanese Women and Men aged 30–76 Years

**DOI:** 10.3390/nu14204270

**Published:** 2022-10-13

**Authors:** Kentaro Murakami, Nana Shinozaki, Nana Kimoto, Shizuko Masayasu, Satoshi Sasaki

**Affiliations:** 1 Department of Social and Preventive Epidemiology, School of Public Health, University of Tokyo, Tokyo 113-0033, Japan; 2 Ikurien-Naka, Ibaraki 311-0105, Japan

**Keywords:** diet, nutrient, questionnaire, food diary, relative validity, Japan

## Abstract

The purpose of this study was to examine the relative validity of the Meal-based Diet History Questionnaire (MDHQ) for estimating nutrient intake. Dietary data were obtained from 111 Japanese women and 111 Japanese men, using the online MDHQ and the 4-non-consecutive-day weighed dietary record (DR). The number of nutrients (total *n* = 46) showing no significant mean differences between estimates from the online MDHQ and DR (with energy adjustment by the density model) was 17 among women and 12 among men. The median value (25th and 75th percentiles) of the Pearson correlation coefficients between the online MDHQ and DR estimates was 0.54 (0.35–0.57) among women and 0.45 (0.25–0.53) among men. Bland–Altman plots for energy-providing nutrients indicated wide limits of agreement (and proportional bias for protein) with overall underestimation of protein and fat and overestimation of carbohydrate by the online MDHQ. Similar results were found when the paper version of the MDHQ (completed after the DR) was examined. For example, the median value of the Pearson correlation coefficients was 0.54 for women and 0.45 for men. This study suggests that the MDHQ has an acceptable ability to rank individuals according to intakes of a wide range of nutrients.

## 1. Introduction

It is broadly recognized that suboptimal dietary intake is a leading modifiable risk factor contributing to both morbidity and early mortality, making the improvement of dietary quality a worldwide priority at present [1]. The accurate evaluation of habitual dietary intake is fundamental to investigating the diet–disease relationship and to facilitating positive changes in dietary behavior [2]. It is often considered that the dietary record (DR) and 24-h dietary recall are the most accurate methods for capturing intakes of a wide variety of foods and nutrients [3,4]. However, assessing habitual dietary intake on an individual level is not always viable because these methods involve the collection of multiple days of dietary data and, despite technological advances, are still burdensome [4,5]. Conversely, dietary assessment questionnaires are the most commonly used tool in large-scale epidemiologic and intervention studies to capture dietary intake [6,7]. Unlike the DR and 24-h dietary recall, dietary assessment questionnaires can capture long-term dietary intake in a single administration and are less cumbersome to complete [8]. However, dietary assessment questionnaires do not necessarily collect information on actual dietary intake but ultimately measure only the memory and perception of usual diet [9]. Another limitation of dietary assessment questionnaires is that participants cannot report foods not included in the questionnaire [4]. Researchers must always find a balance between reducing participant burden and how comprehensive the list of food items should be. Therefore, a successful dietary assessment questionnaire is inevitably a final product of a careful development and validation evaluation process.

As a tool to assess the dietary habits of Japanese people, we recently designed the Meal-based Diet History Questionnaire (MDHQ) [10,11]. There are several features of the MDHQ. First, the MDHQ separately assesses dietary intake for each type of meal (i.e., breakfast, morning snack, lunch, afternoon snack, dinner and evening snack). This is mainly based on previous observations in Japanese adults, in which the selection, amount and combination of foods consumed are markedly different between meal types [12,13,14,15,16]. There are complex cognitive tasks demanded during meal recall, such as understanding the information being sought and searching for and evaluating the retrieved information [17]. Thus, compared with questions asking about overall dietary intake in typical dietary assessment questionnaires, questions arranged for each meal type separately in the MDHQ may be easier to answer, facilitating better estimation of dietary intake [18]. Second, the MDHQ is a data-driven system. That is, the structure of the questionnaire, food items and the development of the dietary intake computation algorithm are based on in-depth dietary information retrieved from the 16-day weighed DR collected from 242 Japanese adults (comprising 206,837 food item entries) [10]. This may also contribute to providing better estimation of dietary intake. Third, the MDHQ, originally developed as a paper self-administered questionnaire [10], can be self-administered online [11]. This feature provides several advantages, including reducing paper use, postage costs and the space, security and organization required for paper file storage [19], as well as the ability to communicate with a geographically dispersed population and groups often difficult to sample [20]. Given that, to our knowledge, only a single online dietary assessment questionnaire is available for the Japanese [21], the MDHQ may be a promising candidate in nutritional epidemiologic research in Japan. Finally, we have recently developed a web-based personalized dietary feedback system that integrates dietary assessment using the MDHQ [11], which might be useful in online intervention trials for promoting favorable changes in dietary behaviors.

However, a rigorous evaluation of the validity of the MDHQ has not been conducted yet, except for food group intake [22]. The main objective of this study was to examine the relative validity of nutrient intake obtained through the web version of the MDHQ (web MDHQ) using the 4-non-consecutive-day weighed DR as a reference. In the real world, not all study participants would complete the questionnaire online. Thus, the secondary objective was to similarly examine the relative validity of the paper version of the MDHQ (paper MDHQ).

## 2. Materials and Methods

### 2.1. Study Procedure and Participants

Details on the survey procedure and participants have been described elsewhere [22]. In brief, a team of research dietitians with expertise in DR data collection (*n* = 66) [23,24] collected data in 14 (of 47) prefectures from August to October 2021. For each prefecture, 8 community-dwelling couples, i.e., 2 women from each of the 4 age categories (30–39, 40–49, 50–59 and 60–69 years) and their husbands (irrespective of age), were recruited. Thus, 112 women and 112 men were invited. Although dietary data from cohabiting couples may reduce gender differences in dietary intake, we chose a priori to separate all analyses into women and men, so we do not consider this issue problematic in this study. We decided our sample size principally based on the recommendations of Cade et al. that for validation studies, a sample size of at least 50 and preferably much larger (e.g., 100 or more subjects) is desirable [4]. Our exclusion criteria were dietitians, people living with a dietitian, people receiving dietary counseling from a physician or dietitian, people with diabetes and receiving insulin therapy, people receiving dialysis treatment, people without adequate access to the Internet, people who had difficulty completing the web-based questionnaire and pregnant or lactating women. Due to snowball sampling, the number of individuals approached for this study and those excluded from the survey were not recorded.

We first asked each participant to complete the web MDHQ. After a 7- to 10-day interval to ensure completion of the web MDHQ, participants were asked to complete the weighed DR on four non-consecutive days within two weeks. Finally, after an interval of at least one day, participants were asked to complete the paper MDHQ. The study protocol was completed by 111 women aged 30 to 69 years and 111 men aged 30 to 76 years. This study was conducted in accordance with the guidelines of the Declaration of Helsinki and all procedures involving humans were approved by the Ethics Committee of the University of Tokyo Faculty of Medicine (protocol code: 2020326NI; date of approval: 29 January 2021). Written informed consent was obtained from all participants.

### 2.2. Meal-based Diet History Questionnaire

The MDHQ has been presented in detail elsewhere [10,11]. In short, the MDHQ is a self-administered questionnaire that is designed to assess dietary intake in the previous month for each meal type (breakfast, morning snack, lunch, afternoon snack, dinner and night snack). The MDHQ is comprised of three parts. In Part 1 of the MDHQ, quantitative questions regarding the frequency of consumption of common food groups (i.e., Tier 1 food groups) for each meal type are included (*n* = 11–24 for common food groups depending on meal type), which can be answered from 0 to 7 days/week. In Part 2 of the MDHQ, there are questions about the relative frequency of consumption of sub-food groups within Tier 1 food groups (i.e., Tier 2 food groups; *n* = 0–19 according to Tier 1 food groups), which can be answered as “always”, “often”, “sometimes”, “rarely”, or “never”. The information obtained from Part 1 and Part 2 can be combined to increase the number of foods that can be effectively estimated within a restricted number of questions. In Part 3 of the MDHQ, the respondents are asked about general eating behaviors, such as the relative frequency of consumption of brown rice and whole grain bread and whether they eat their bread with jam, honey, or spread fat on it. At the end, the MDHQ involves an assessment of basic attributes (i.e., sex, age, height, weight, education level and current smoking status).

The MDHQ does not gather information on portion sizes (except for alcoholic beverages for which overall consumption frequency and portion sizes are assessed in Part 2). Our rationale for this decision was rooted in our previous finding: a simplified diet history questionnaire (brief-type diet history questionnaire, BDHQ), which assesses the frequency of consumption of 58 food items but collects no information on portion size and applies fixed portion sizes in the calculation of dietary intake, is as effective in estimating food and nutrient intake as a comprehensive diet history questionnaire (DHQ), which not only assesses frequency of consumption but also portion size for 150 food items [25,26,27]. Several previous studies support the limited utility of portion size information [28,29]. All the food groups in the MDHQ and sex-specific and meal-type specific fixed portion size were derived from the 16-day weighed DR data collected from 242 Japanese adults [10].

Two delivery modes of the MDHQ used in this study (web MDHQ and paper MDHQ) were identical in terms of content. The web MDHQ was created using Google Forms. Every question was answered by each participant; no non-responses were allowed. All responses to the web MDHQ, which were automatically allocated to a spreadsheet format, were downloaded from Google Drive. The paper MDHQ utilized in this study was a 21-page A4 questionnaire. The responses to all questions were verified by the research dietitians and the research center staff. Where answers were missing, we asked participants to re-answer the questions in person or over the phone. All responses to the paper MDHQ were typed by hand in duplicate on a spreadsheet and any inconsistencies were checked and corrected. The data obtained using the web and paper versions of the MDHQ were then transformed into a dataset that was suitable for dietary intake calculations.

On the basis of a series of ad hoc computer algorithms in the MDHQ [10], estimated intakes of Tier 1 and 2 food groups were calculated. Estimated intakes of energy and nutrients were calculated using food intake information and the 2015 version of the Standard Tables of Food Composition in Japan [30]. For food items with unavailable data on the added sugar content, added sugar values were calculated based on the same or similar food in the 2011–2012 Food Patterns Equivalents Database [31]. Teaspoon equivalents in the Food Patterns Equivalents Database were converted into grams by multiplying by 4.2 (grams of added sugar per teaspoon). The number of food codes used during the calculations was 763 for breakfast, 909 for lunch, 965 for dinner, 695 for morning snack and 703 for afternoon and night snacks [10]. The calculation was made for each meal type and the overall intake was calculated as the sum of the intake of each meal type.

### 2.3. Weighed Dietary Record

Details on the 4-non-consecutive-day weighed DR used as the reference method in this validation study have been presented elsewhere [22]. Briefly, each recording period comprised three weekdays (Monday to Friday) and one weekend day (Saturday or Sunday) within two weeks. Each couple was issued recording sheets and a digital scale (KS-274, Dretec, Japan; ±2 g accuracy for 0–500 g, ±3 g accuracy for 500–2000 g). Upon receiving written and verbal instructions from the research dietitian and a sample diary entry, each participant was asked to document and weigh everything they ate and drank, both inside and outside the home, for each day of record keeping. In cases where weighing is difficult, such as eating out, the participants were instructed to record as much information as possible, including the brand name of the food, the amount consumed (using typical household scales) and the contents of leftovers.

The recording forms used for each survey day were submitted directly to the research dietitian after the survey was completed, who reviewed the forms and, if necessary, requested additional information or revised records by telephone or in person. All records collected were checked by the research dietitians and trained staff at the study center. Following the standard procedure, estimated portions by using a household scale were transformed into weights and individual food items were coded according to the 2015 version of the Standard Tables of Food Composition of Japan [30]. In total, 1297 food codes were used in the DR. As in the MDHQ, estimated energy and nutrient intakes were calculated using the 2015 version of the Standard Tables of Food Composition of Japan [30] and the 2011–2012 Food Pattern Equivalents Database [31]. We used the average daily value for each individual over the four-day period for all dietary variables.

### 2.4. Statistical Analysis

Statistical analyses were performed using the SAS statistical software (version 9.4; SAS Institute Inc., Cary, NC, USA). A two-tailed *p* value of <0.05 was considered significant. All analyses were conducted for women and men separately. The dietary variables examined in this study included 46 nutrients. Dietary data were expressed as mean and standard deviation (SD). Analyses were conducted using energy-adjusted values by the residual and density models [32], as well as using crude values. In this study, nutrient intake from dietary supplements was not considered, mainly because of a lack of reliable food composition database in Japan.

To assess the estimation ability at the group level, the mean values of estimates derived from the MDHQ were compared with those derived from the DR using the paired *t*-test. In order to assess the ability of the MDHQ to rank individuals in the population, Pearson correlation coefficients between the MDHQ and DR estimates were used. Additionally, the agreement between the MDHQ and DR for protein, fat and carbohydrate estimates from the density model (% of energy) was evaluated using Bland–Altman plots [33]. We also used linear regression analysis to examine proportional bias between the MDHQ and DR [34]. We performed identical analyses to evaluate the web MDHQ and paper MDHQ; we provide our findings for the web MDHQ in the Section 3 and for the paper MDHQ in the Supplementary Materials.

## 3. Results

Table 1 shows the basic characteristics of the study subjects. The mean body mass index (BMI) values (kg/m^2^) were 22.7 (SD: 3.3) for women and 23.8 (SD: 3.6) for men. For both women and men, the mean energy intakes estimated from the web MDHQ and paper MDHQ were significantly (*p* < 0.001) lower than that estimated from the DR.

### 3.1. Results on the Web Version of Meal-Based Diet History Questionnaire

#### 3.1.1. Mean Estimation

The mean estimates of energy-adjusted intakes of nutrients derived from the DR and web MDHQ are shown in Table 2 for women and Table 3 for men. Among women, the number of nutrients (*n* = 46 in total) that showed no significant mean differences between the web MDHQ and DR estimates was 12 (26%) for the residual model and 17 (37%) for the density model. The corresponding value among men was 7 (15%) and 13 (28%), respectively. When the crude values were examined (Table S1), the results were similar to those based on the residual model; the corresponding value was 12 (26%) among women and 7 (15%) among men.

#### 3.1.2. Pearson Correlations

Table 4 shows the Pearson correlation coefficients between crude and energy-adjusted estimates of daily intakes of nutrients derived from the DR and web MDHQ. For crude estimates of nutrient intakes, the median values of the Pearson correlation coefficients (25th and 75th percentiles) were 0.37 (0.30–0.46) among women and 0.33 (0.21–0.40) among men. Higher correlations were observed when energy-adjusted values of nutrient intakes were examined than when using crude estimates, particularly among women. The median values of the Pearson correlation coefficients (25th and 75th percentiles) among women were 0.50 (0.34–0.56) for the residual model and 0.54 (0.35–0.57) for the density model. The corresponding values among men were 0.37 (0.26–0.49) and 0.45 (0.25–0.53), respectively.

#### 3.1.3. Bland–Altman Plots

**Figure 1:**
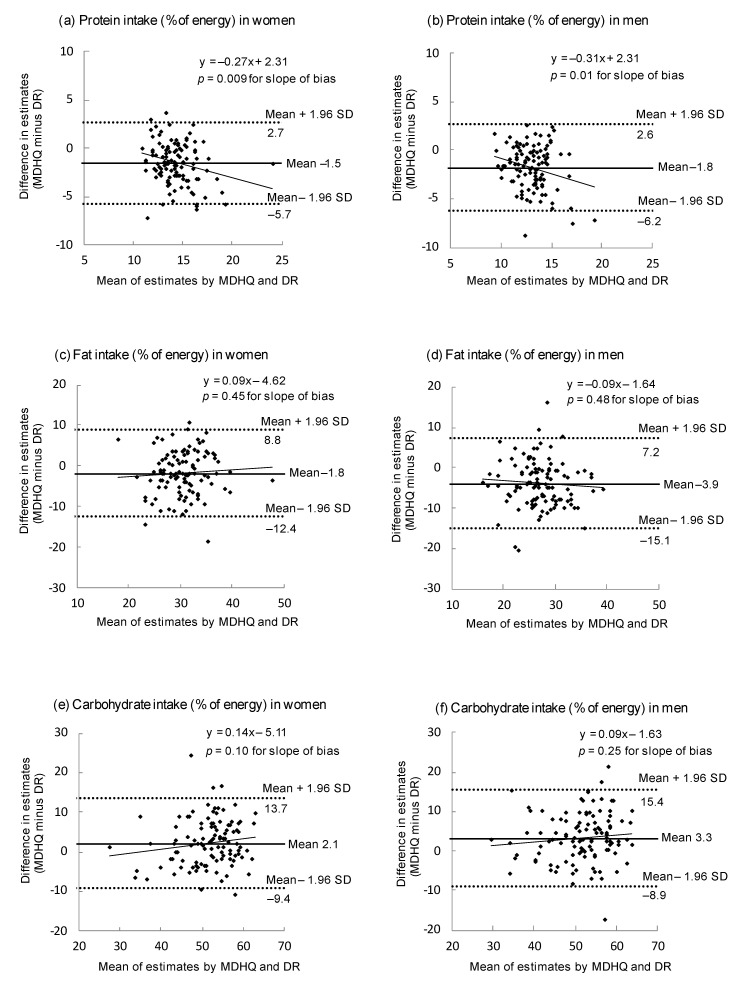
Bland–Altman plots assessing the agreement between estimates of energy-adjusted intakes of protein (**a**,**b**), fat (**c**,**d**) and carbohydrate (**e**,**f**) obtained from the 4-day weighed dietary record (DR) and those obtained from the web version of the Meal-based Diet History Questionnaire (MDHQ) in 111 Japanese women and 111 Japanese men. Energy adjustment was made using the density model. SD, standard deviation.

Figure 1 shows Bland–Altman plots assessing the agreement between estimates of energy-adjusted intakes of protein, fat and carbohydrate (% of energy) derived from the DR and those derived from the web MDHQ. Overall, compared to the DR, the web MDHQ underestimated protein and fat intakes, with a range from 1.5% of energy (for protein in women) to 3.9% of energy (for fat in men). Conversely, carbohydrate intake was overestimated by the web MDHQ compared with the DR, by 2.1% of energy among women and 3.3% of energy among men. Further, regardless of sex and nutrient, the limits of agreement (mean difference ± 1.96 SD of the difference) were generally wide, indicating poor agreement at the individual level. There was no indication of proportional bias between the web MDHQ and DR, except for protein intake which tended to be underestimated by the web MDHQ as the average intake increased in both sexes.

### 3.2. Results from the Paper Version of Meal-Based Diet History Questionnaire

Identical analyses of the paper MDHQ were conducted (Tables S2 and S3 for mean estimations, Table S4 for Pearson correlation coefficients and Figure S1 for Bland–Altman plots for protein, fat and carbohydrate intakes). The results for the paper MDHQ were generally similar to those for the web MDHQ, except for somewhat higher Pearson correlation coefficients between the paper MDHQ and DR, particularly in the crude and residual models. The median values of the Pearson correlation coefficients (25th and 75th percentiles) among women were 0.45 (0.38–0.49) for the crude model, 0.56 (0.46–0.61) for the residual model and 0.54 (0.42–0.59) for the density model. The corresponding values among men were 0.42 (0.35–0.50), 0.43 (0.34–0.55) and 0.45 (0.32–0.57), respectively.

## 4. Discussion

As a companion paper on the relative validity of the MDHQ at the food level [22], here we examined the relative validity of the MDHQ at the nutrient level, using the same dataset. We observed that, for many nutrients (63% to 85% of nutrients examined), energy-adjusted mean values derived from the web MDHQ were significantly different from those derived from the 4-day weighed DR, irrespective of energy adjustment model and sex. These findings suggest that the web MDHQ is acceptable for estimating mean values for only a limited number of nutrients. Similar results (50% to 90% of nutrients examined showing significant differences) have been obtained in previous relative validation analyses of the DHQ and BDHQ, which are among the most widely used dietary assessment questionnaires in Japan [26].

Additionally, on the basis of Bland–Altman plots for energy-providing nutrients, we found poor agreement between the web MDHQ and DR at the individual level, which is consistent with our analysis of the web MDHQ for food groups [22]. This may be mainly due to the use of the fixed portion sizes during dietary intake calculation. In any case, irrespective of energy adjustment, the estimates of nutrient intakes derived from the web MDHQ should be interpreted with considerable caution not only at the individual level but also as at the group level.

Nevertheless, we observed that, for many nutrients, the Pearson correlation coefficients between energy-adjusted estimates derived from the web MDHQ and DR were greater than 0.40 in both women (70% for the residual model and 67% for the density model) and men (43% for the residual model and 57% for the density model). These findings suggest that the web MDHQ has an acceptable ability to rank individuals according to intakes of a wide range of nutrients. Somewhat better results (57% to 83% of nutrients examined) have been obtained in previous validation analyses of the DHQ and BDHQ using the 16-day DR as a reference [26].

For ranking ability of dietary assessment questionnaires in Japan, a review published in 2009 showed that the median of correlation coefficients between dietary assessment questionnaires and DR ranged from 0.31 to 0.56 [35]. Similar results have been reported in more recent studies, with a range of median of correlation coefficients from 0.44 to 0.52 [21,26,36,37]. Particularly, in a validation study on intakes of energy and 53 nutrients derived from an online food frequency questionnaire against a 12-day DR, the median value of correlation coefficients was 0.46 for women and 0.47 for men [21]. The median values of the Pearson correlation coefficients observed in this study (0.37 to 0.54, depending on sex and energy adjustment model) are comparable with these figures. Collectively, we consider that the web MDHQ’s ability for ranking individuals according to nutrient intake is not inferior to that of existing dietary assessment questionnaires in Japan, as in the case of food intake estimation [22].

However, being “non-inferior” to other questionnaires does not necessarily justify the use of the web MDHQ. For example, we observed that the correlation coefficients were rather low (<0.30) for such nutrients as n-3 polyunsaturated fatty acid (only men), marine-origin n-3 fatty acids (including individual fatty acids; only men), α-linolenic acid, water, retinol, cryptoxanthin, retinol equivalent, vitamin D (only men), niacin (only men) and vitamin B-12 (only men), which may be mainly due to the limited validity of major food sources of these nutrients (e.g., fish, vegetable oils and meat) [22]. The low correlations may also be due to high within-person variability in intakes of these nutrients [38]. Most of these nutrients are fat-related and difficulty in assessing intakes of these nutrients has also been observed in a recent meta-analysis of correlation coefficients between food frequency questionnaires and reference methods (DR or 24-h dietary recall) [3]. Conversely, we observed that the correlation coefficients were rather high (>0.60) for some nutrients, including marine-origin n-3 polyunsaturated fatty acids (only women), carbohydrate, alcohol, pantothenic acid (only women), vitamin C (only men), sodium (only women), potassium, magnesium (only women), iron and copper (only women), which may be mainly due to a high validity of major food sources of these nutrients (e.g., rice, miso soup, fruit, dairy products and alcoholic beverages) [22]. These findings should be carefully considered when selecting the web MDHQ (or another questionnaire) as a dietary assessment tool in nutrition research in Japan.

It should be noted that the analyses based on the web MDHQ and on the paper MDHQ showed similar results, despite the fact that the Pearson correlation coefficients with the DR were somewhat high for the paper MDHQ, as was observed in the validation analysis of food group intake [22]. Apparently higher correlations for the paper MDHQ are reasonable since the web MDHQ and paper MDHQ are filled out before and after the experience of dietary recording, respectively. In general, administrative and cost considerations favor online surveys, but not all research participants will cooperate with online surveys in the real world. Therefore, we plan to directly compare the food and nutrient intakes provided by the web and paper versions of MDHQ to evaluate the comparability or compatibility of these two modes of delivery.

Several limitations in the present study have been described in detail elsewhere [22]; thus, a brief description is provided here. First, the subjects of this study are not a nationally representative sample of the Japanese population and may be biased toward those who are more health conscious. Thus, further research needs to be conducted with a more representative sample. Second, the reference method of this study, weighed DR, is not without measurement error, especially because of incorrect recording and potential changes in eating behavior [4]. However, the weighed DR is broadly regarded as the first method for validating diet assessment questionnaires since the errors in weighed DR should be less correlated with the errors in dietary assessment questionnaires than the errors in other memory-based diet assessment tools [3,4]. Nevertheless, we cannot rule out the possibility that the weighed DR and MDHQ share sources of bias, which could affect the present results, including the calculation of correlation coefficients. Finally, because the MDHQ is designed to assess dietary habits during the previous month, the tool does not account for intra-individual variation in dietary intake during the year (e.g., changes in dietary intake for the same person due to seasonal or temporal differences in foods). However, our earlier work has demonstrated that a one-time administering of questionnaires which assess dietary intake during the preceding month (i.e., DHQ and BDHQ) may be able to capture longer-term (i.e., one year) habitual dietary intake [25,26,27,39] and these results may also be extrapolated to the MDHQ.

In summary, consistent with the analysis at the food group level [22], we showed that the web version (as well as the paper version) of the MDHQ had acceptable relative validity in terms of nutrient intakes against the 4-day weighed DR. For a wide range of nutrients, the MDHQ’s ability to rank individuals was acceptable, whereas its ability to estimate mean intakes appeared limited for only a small number of nutrients, as well as a limited ability to estimate nutrient intakes at the individual level. To our knowledge, there is no purpose-built, dedicated dietary assessment questionnaire to collect data on dietary intake at each meal type, which is also inexpensive to implement and less burdensome for participants. Thus, we consider that the MDHQ, a novel, purpose-built, dedicated dietary assessment questionnaire to collect data on dietary intake at each meal type, might be useful for future nutritional epidemiologic research. In this context, this analysis of nutrient intakes, as well as that on food group intakes [22], might lend support to the use of the MDHQ in large-scale epidemiologic and intervention studies in Japan to capture dietary intake.

## Figures and Tables

**Table 1:** Basic characteristics of the study subjects ^1^.

Variable	Women (*n* = 111)	Men (*n* = 111)
Age (years)	49.9 ± 10.7	51.7 ± 11.9
Body height (cm) ^2^	158.4 ± 5.4	170.2 ± 6.3
Body weight (kg) ^2^	56.9 ± 8.5	68.9 ± 11.9
Body mass index (kg/m^2^) ^3^	22.7 ± 3.3	23.8 ± 3.6
Education level (*n* (%))		
Junior high school or high school	28 (25.2)	41 (36.9)
College or technical school	55 (49.5)	22 (19.8)
University or higher	28 (25.2)	48 (43.2)
Current smoking status (*n* (%))		
Smoker	12 (10.8)	35 (31.5)
Nonsmoker	99 (89.2)	76 (68.4)
Energy intake (kcal/day)		
4-day DR	1724 ± 335	2286 ± 493
Web version of MDHQ	1470 ± 349	1926 ± 517
Paper version of MDHQ	1509 ± 320	1895 ± 420

Abbreviations: DR, dietary record; MDHQ, Meal-based Diet History Questionnaire. ^1^ Values are expressed as mean ± standard deviation, unless otherwise indicated. ^2^ Based on self-report. ^3^ Calculated using the self-reported body height and weight.

**Table 2:** Mean estimates of energy-adjusted intakes of nutrients obtained from the 4-day weighed dietary record (DR) and those obtained from the web version of the Meal-based Diet History Questionnaire (MDHQ) in 111 Japanese women ^1^.

	Residual Model			Density Model		
	Unit	DR	Web MDHQ	Unit	DR	Web MDHQ
Protein	g/day	64.0 ± 11.1	48.9 ± 7.0 c	% of energy	15.0 ± 2.5	13.4 ± 2.0 c
Fat	g/day	60.2 ± 10.4	48.5 ± 8.3 c	% of energy	31.4 ± 4.9	29.6 ± 5.2 c
SFA	g/day	18.0 ± 3.9	14.9 ± 3.5 c	% of energy	9.4 ± 2.0	9.0 ± 2.1
MUFA	g/day	22.9 ± 5.1	17.6 ± 3.2 c	% of energy	11.9 ± 2.3	10.8 ± 2.0 c
PUFA	g/day	12.2 ± 3.1	10.7 ± 2.2 c	% of energy	6.4 ± 1.5	6.6 ± 1.3
n-6 PUFA	g/day	10.1 ± 2.6	9.0 ± 1.7 c	% of energy	4.1 ± 1.0	5.5 ± 1.1 c
n-3 PUFA	g/day	2.13 ± 0.89	1.74 ± 0.52 c	% of energy	0.87 ± 0.34	1.07 ± 0.36 c
Marine-origin n-3 PUFA ^2^	g/day	0.66 ± 0.59	0.41 ± 0.26 c	% of energy	0.27 ± 0.22	0.26 ± 0.21
EPA	g/day	0.21 ± 0.22	0.13 ± 0.09 c	% of energy	0.09 ± 0.08	0.08 ± 0.07
n-3 DPA	g/day	0.07 ± 0.05	0.04 ± 0.02 c	% of energy	0.03 ± 0.02	0.03 ± 0.02
DHA	g/day	0.38 ± 0.33	0.24 ± 0.15 c	% of energy	0.16 ± 0.12	0.15 ± 0.12
α-linolenic acid	g/day	1.38 ± 0.65	1.28 ± 0.31	% of energy	0.56 ± 0.27	0.78 ± 0.19 c
Cholesterol	mg/day	302 ± 97	228 ± 65 c	mg/1000 kcal	177 ± 57	155 ± 46 c
Carbohydrate	g/day	217.2 ± 32.6	193.1 ± 29.9 c	% of energy	50.5 ± 6.7	52.7 ± 7.5 c
Added sugars	g/day	30.3 ± 15.5	31.0 ± 17.0	% of energy	6.9 ± 3.4	8.2 ± 4.6 b
Soluble dietary fiber	g/day	2.94 ± 0.90	2.16 ± 0.48 c	g/1000 kcal	1.72 ± 0.51	1.47 ± 0.33 c
Insoluble dietary fiber	g/day	8.93 ± 2.89	6.98 ± 1.49 c	g/1000 kcal	5.16 ± 1.49	4.76 ± 0.99 c
Total dietary fiber	g/day	12.3 ± 3.7	9.8 ± 2.2 c	g/1000 kcal	7.1 ± 1.9	6.7 ± 1.5 b
Alcohol	g/day	5.67 ± 13.59	7.37 ± 15.96	% of energy	2.08 ± 4.50	3.29 ± 6.74 b
Water	g/day	2214 ± 565	2329 ± 518	g/1000 kcal	1307 ± 384	1618 ± 418 c
Retinol	μg/day	188 ± 346	133 ± 50	μg/1000 kcal	110 ± 203	91 ± 34
α-carotene	μg/day	489 ± 454	512 ± 347	μg/1000 kcal	286 ± 288	349 ± 241 a
β-carotene	μg/day	2322 ± 1430	2266 ± 1156	μg/1000 kcal	1335 ± 853	1533 ± 780 b
Cryptoxanthin	μg/day	92.5 ± 182.2	124.8 ± 190.0	μg/1000 kcal	52.0 ± 98.4	86.3 ± 131.3 a
β-carotene equivalent ^3^	μg/day	2648 ± 1634	2620 ± 1301	μg/1000 kcal	1524 ± 985	1775 ± 881 b
Retinol equivalent ^4^	μg/day	409 ± 378	353 ± 115	μg/1000 kcal	237 ± 222	240 ± 80
Vitamin D	μg/day	5.91 ± 4.26	4.23 ± 2.05 c	μg/1000 kcal	3.45 ± 2.30	2.90 ± 1.60 b
α-tocopherol	mg/day	6.89 ± 1.57	5.73 ± 1.28 c	mg/1000 kcal	4.01 ± 0.88	3.88 ± 0.90
Vitamin K	μg/day	195.2 ± 91.9	162.5 ± 67.9 c	μg/1000 kcal	112.6 ± 50.4	111.4 ± 46.8
Thiamin	mg/day	0.86 ± 0.25	0.70 ± 0.13 c	mg/1000 kcal	0.50 ± 0.13	0.48 ± 0.09 a
Riboflavin	mg/day	1.12 ± 0.26	0.95 ± 0.17 c	mg/1000 kcal	0.66 ± 0.15	0.65 ± 0.13
Niacin	mg/day	16.2 ± 4.3	13.3 ± 2.8 c	mg/1000 kcal	9.5 ± 2.4	9.2 ± 2.1
Vitamin B-6	mg/day	1.13 ± 0.34	0.87 ± 0.20 c	mg/1000 kcal	0.65 ± 0.18	0.60 ± 0.14 c
Vitamin B-12	μg/day	4.88 ± 3.14	3.38 ± 1.76 c	μg/1000 kcal	2.83 ± 1.61	2.36 ± 1.55 b
Folate	μg/day	270 ± 87	220 ± 55 c	μg/1000 kcal	158 ± 48	150 ± 37
Pantothenic acid	mg/day	5.18 ± 0.92	4.20 ± 0.62 c	mg/1000 kcal	3.03 ± 0.56	2.87 ± 0.42 c
Vitamin C	mg/day	79 ± 32	68 ± 23 c	mg/1000 kcal	46 ± 19	46 ± 16
Sodium	mg/day	3340 ± 782	3221 ± 711	mg/1000 kcal	1937 ± 432	2206 ± 498 c
Potassium	mg/day	2242 ± 515	1968 ± 380 c	mg/1000 kcal	1307 ± 299	1351 ± 272
Calcium	mg/day	477 ± 170	415 ± 106 c	mg/1000 kcal	277 ± 96	285 ± 75
Magnesium	mg/day	244 ± 56	219 ± 43 c	mg/1000 kcal	141 ± 32	150 ± 30 c
Phosphorus	mg/day	951 ± 174	774 ± 120 c	mg/1000 kcal	555 ± 100	531 ± 84 b
Iron	mg/day	6.72 ± 1.73	5.41 ± 1.06 c	mg/1000 kcal	3.91 ± 0.95	3.70 ± 0.74 b
Zinc	mg/day	7.56 ± 1.31	6.10 ± 0.78 c	mg/1000 kcal	4.40 ± 0.77	4.19 ± 0.54 b
Copper	mg/day	1.00 ± 0.24	0.84 ± 0.14 c	mg/1000 kcal	0.58 ± 0.12	0.58 ± 0.09
Manganese	mg/day	2.86 ± 0.89	2.85 ± 1.13	mg/1000 kcal	1.68 ± 0.53	1.97 ± 0.72 c

Abbreviations: SFA, saturated fatty acids; MUFA, monounsaturated fatty acids; PUFA, polyunsaturated fatty acids; EPA, eicosapentaenoic acid; DPA, Docosapentaenoic acid; DHA, Docosahexaenoic acid. ^1^ Values are means ± standard deviations. The values derived from the MDHQ were compared with those derived from the DR using the paired *t*-test: a, *p* < 0.05; b, *p* < 0.01; c, *p* < 0.001. ^2^ Sum of EPA, n-3 DPA and DHA. ^3^ Sum of β-carotene, α-carotene/2 and cryptoxanthin/2. ^4^ Sum of retinol, β-carotene/12, α-carotene/24 and cryptoxanthin/24.

**Table 3:** Mean estimates of energy-adjusted intakes of nutrients obtained from the 4-day weighed dietary record (DR) and those obtained from the web version of the Meal-based Diet History Questionnaire (MDHQ) in 111 Japanese men ^1^.

	Residual Model			Density Model		
	Unit	DR	Web MDHQ	Unit	DR	Web MDHQ
Protein	g/day	79.0 ± 11.8	57.9 ± 8.6 c	% of energy	14.0 ± 2.2	12.2 ± 1.8 c
Fat	g/day	72.9 ± 14.8	53.1 ± 12.1 c	% of energy	28.9 ± 5.5	25.0 ± 5.2 c
SFA	g/day	20.9 ± 5.1	15.7 ± 5.0 c	% of energy	8.3 ± 1.9	7.3 ± 2.0 c
MUFA	g/day	28.5 ± 6.7	19.8 ± 4.4 c	% of energy	11.3 ± 2.5	9.3 ± 2.0 c
PUFA	g/day	14.9 ± 3.9	11.8 ± 2.8 c	% of energy	5.9 ± 1.5	5.6 ± 1.4
n-6 PUFA	g/day	12.2 ± 3.3	9.8 ± 2.3 c	% of energy	3.7 ± 1.0	4.7 ± 1.1 c
n-3 PUFA	g/day	2.71 ± 1.15	1.98 ± 0.58 c	% of energy	0.84 ± 0.37	0.95 ± 0.29 b
Marine-origin n-3 PUFA ^2^	g/day	0.87 ± 0.64	0.55 ± 0.27 c	% of energy	0.27 ± 0.20	0.26 ± 0.13
EPA	g/day	0.28 ± 0.23	0.17 ± 0.09 c	% of energy	0.09 ± 0.07	0.08 ± 0.05
n-3 DPA	g/day	0.09 ± 0.06	0.06 ± 0.02 c	% of energy	0.03 ± 0.02	0.03 ± 0.01
DHA	g/day	0.50 ± 0.36	0.32 ± 0.15 c	% of energy	0.15 ± 0.11	0.15 ± 0.08
α-linolenic acid	g/day	1.73 ± 0.92	1.36 ± 0.41 c	% of energy	0.54 ± 0.30	0.65 ± 0.20 c
Cholesterol	mg/day	378 ± 112	257 ± 86 c	mg/1000 kcal	168 ± 53	134 ± 45 c
Carbohydrate	g/day	282.1 ± 41.9	252.9 ± 38.7 c	% of energy	49.8 ± 7.5	53.1 ± 8.2 c
Added sugars	g/day	35.5 ± 27.1	33.4 ± 22.5	% of energy	6.3 ± 4.9	6.8 ± 4.4
Soluble dietary fiber	g/day	3.24 ± 0.91	2.34 ± 0.66 c	g/1000 kcal	1.43 ± 0.44	1.22 ± 0.36 c
Insoluble dietary fiber	g/day	9.73 ± 2.65	7.72 ± 1.94 c	g/1000 kcal	4.29 ± 1.13	4.07 ± 1.01 a
Total dietary fiber	g/day	13.4 ± 3.5	10.7 ± 2.8 c	g/1000 kcal	5.9 ± 1.5	5.6 ± 1.5
Alcohol	g/day	21.75 ± 26.59	25.33 ± 29.31	% of energy	5.82 ± 7.99	8.27 ± 9.85 c
Water	g/day	2683 ± 566	2642 ± 772	g/1000 kcal	1179 ± 247	1397 ± 419 c
Retinol	μg/day	247 ± 570	132 ± 72 a	μg/1000 kcal	114 ± 282	67 ± 35
α-carotene	μg/day	515 ± 477	573 ± 454	μg/1000 kcal	229 ± 217	307 ± 248 b
β-carotene	μg/day	2437 ± 1439	2265 ± 1344	μg/1000 kcal	1074 ± 656	1206 ± 725 a
Cryptoxanthin	μg/day	66.6 ± 106.6	111.9 ± 167.2 a	μg/1000 kcal	29.6 ± 49.0	56.6 ± 80.8 b
β-carotene equivalent ^3^	μg/day	2770 ± 1654	2644 ± 1557	μg/1000 kcal	1223 ± 755	1405 ± 841 a
Retinol equivalent ^4^	μg/day	479 ± 597	354 ± 157 a	μg/1000 kcal	217 ± 291	186 ± 81
Vitamin D	μg/day	7.04 ± 4.59	5.29 ± 2.22 c	μg/1000 kcal	3.09 ± 2.00	2.81 ± 1.25
α-tocopherol	mg/day	8.22 ± 2.11	6.20 ± 1.64 c	mg/1000 kcal	3.61 ± 0.89	3.26 ± 0.87 c
Vitamin K	μg/day	208.6 ± 100.8	155.7 ± 80.8 c	μg/1000 kcal	92.1 ± 43.2	82.7 ± 42.6 a
Thiamin	mg/day	1.05 ± 0.23	0.81 ± 0.17 c	mg/1000 kcal	0.46 ± 0.10	0.43 ± 0.09 c
Riboflavin	mg/day	1.31 ± 0.32	1.03 ± 0.25 c	mg/1000 kcal	0.58 ± 0.15	0.54 ± 0.13 c
Niacin	mg/day	20.7 ± 4.7	16.7 ± 3.7 c	mg/1000 kcal	9.1 ± 2.2	8.9 ± 2.2
Vitamin B-6	mg/day	1.38 ± 0.34	1.05 ± 0.28 c	mg/1000 kcal	0.61 ± 0.15	0.55 ± 0.14 c
Vitamin B-12	μg/day	5.93 ± 3.25	4.24 ± 1.66 c	μg/1000 kcal	2.62 ± 1.50	2.23 ± 0.88 b
Folate	μg/day	304 ± 105	237 ± 71 c	μg/1000 kcal	135 ± 49	125 ± 41 a
Pantothenic acid	mg/day	6.20 ± 1.16	4.81 ± 0.89 c	mg/1000 kcal	2.73 ± 0.54	2.52 ± 0.43 c
Vitamin C	mg/day	90 ± 37	70 ± 30 c	mg/1000 kcal	40 ± 17	37 ± 17 a
Sodium	mg/day	4335 ± 1167	3931 ± 893 c	mg/1000 kcal	1900 ± 490	2085 ± 530 c
Potassium	mg/day	2536 ± 545	2146 ± 467 c	mg/1000 kcal	1119 ± 246	1131 ± 252
Calcium	mg/day	505 ± 188	399 ± 131 c	mg/1000 kcal	222 ± 85	205 ± 62 a
Magnesium	mg/day	281 ± 64	247 ± 51 c	mg/1000 kcal	123 ± 26	129 ± 26 b
Phosphorus	mg/day	1127 ± 209	883 ± 178 c	mg/1000 kcal	497 ± 93	461 ± 79 c
Iron	mg/day	7.70 ± 1.68	5.96 ± 1.30 c	mg/1000 kcal	3.40 ± 0.74	3.14 ± 0.71 c
Zinc	mg/day	9.20 ± 1.48	7.23 ± 1.23 c	mg/1000 kcal	4.06 ± 0.66	3.81 ± 0.59 c
Copper	mg/day	1.18 ± 0.22	0.99 ± 0.17 c	mg/1000 kcal	0.52 ± 0.09	0.52 ± 0.08
Manganese	mg/day	3.56 ± 1.16	3.42 ± 1.39	mg/1000 kcal	1.58 ± 0.52	1.81 ± 0.67 c

Abbreviations: SFA, saturated fatty acids; MUFA, monounsaturated fatty acids; PUFA, polyunsaturated fatty acids; EPA, eicosapentaenoic acid; DPA, Docosapentaenoic acid; DHA, Docosahexaenoic acid. ^1^ Values are means ± standard deviations. The values derived from the MDHQ were compared with those derived from the DR using the paired *t*-test: a, *p* < 0.05; b, *p* < 0.01; c, *p* < 0.001. ^2^ Sum of EPA, n-3 DPA and DHA. ^3^ Sum of β-carotene, α-carotene/2 and cryptoxanthin/2. ^4^ Sum of retinol, β-carotene/12, α-carotene/24 and cryptoxanthin/24.

**Table 4:** Pearson correlation coefficients between crude and energy-adjusted estimates of daily intakes of nutrients obtained from the 4-day weighed dietary record and those obtained from the web version of the Meal-based Diet History Questionnaire in 111 Japanese women and 111 Japanese men ^1^.

		Women			Men	
	Crude Model	Residual Model	Density Model	Crude Model	Residual Model	Density Model
Protein	0.34 c	0.48 c	0.55 c	0.29 b	0.31 c	0.40 c
Fat	0.27 b	0.43 c	0.43 c	0.07	0.30 b	0.44 c
SFA	0.34 c	0.49 c	0.46 c	0.15	0.40 c	0.45 c
MUFA	0.20 a	0.33 c	0.34 c	0.05	0.26 b	0.39 c
PUFA	0.26 b	0.30 b	0.35 c	0.11	0.22 a	0.40 c
n-6 PUFA	0.22 a	0.27 b	0.30 b	0.10	0.22 a	0.40 c
n-3 PUFA	0.36 c	0.34 c	0.36 c	0.14	0.18	0.21 a
Marine-origin n-3 PUFA ^2^	0.63 c	0.62 c	0.55 c	0.33 c	0.31 b	0.25 b
EPA	0.62 c	0.60 c	0.55 c	0.34 c	0.32 c	0.26 b
n-3 DPA	0.53 c	0.54 c	0.45 c	0.26 b	0.24 a	0.21 a
DHA	0.64 c	0.62 c	0.55 c	0.32 c	0.29 b	0.23 a
α-linolenic acid	0.07	−0.07	−0.01	−0.05	0.03	0.12
Cholesterol	0.44 c	0.43 c	0.38 c	0.50 c	0.46 c	0.45 c
Carbohydrate	0.53 c	0.67 c	0.67 c	0.44 c	0.66 c	0.69 c
Added sugars	0.29 b	0.28 b	0.32 c	0.17	0.28 b	0.35 c
Soluble dietary fiber	0.35 c	0.53 c	0.54 c	0.29 b	0.45 c	0.47 c
Insoluble dietary fiber	0.29 b	0.48 c	0.56 c	0.37 c	0.57 c	0.60 c
Total dietary fiber	0.31 b	0.52 c	0.59 c	0.36 c	0.57 c	0.59 c
Alcohol	0.75 c	0.73 c	0.79 c	0.73 c	0.61 c	0.83 c
Water	0.45 c	0.31 b	0.28 b	0.35 c	0.22 a	0.23 a
Retinol	0.12	0.12	0.11	0.12	0.15	0.15
α-carotene	0.42 c	0.46 c	0.46 c	0.42 c	0.43 c	0.42 c
β-carotene	0.43 c	0.55 c	0.56 c	0.50 c	0.54 c	0.51 c
Cryptoxanthin	0.06	0.07	0.03	0.07	0.05	0.12
β-carotene equivalent ^3^	0.44 c	0.56 c	0.55 c	0.48 c	0.52 c	0.49 c
Retinol equivalent ^4^	0.24 a	0.28 b	0.29 b	0.24 a	0.26 b	0.25 b
Vitamin D	0.46 c	0.51 c	0.49 c	0.18	0.20 a	0.21 a
α-tocopherol	0.21 a	0.34 c	0.33 c	0.22 a	0.36 c	0.45 c
Vitamin K	0.46 c	0.54 c	0.57 c	0.34 c	0.39 c	0.49 c
Thiamin	0.22 a	0.28 b	0.35 c	0.26 b	0.37 c	0.41 c
Riboflavin	0.45 c	0.57 c	0.58 c	0.36 c	0.46 c	0.51 c
Niacin	0.37 c	0.35 c	0.32 c	0.34 c	0.15	0.18
Vitamin B-6	0.34 c	0.43 c	0.53 c	0.46 c	0.38 c	0.37 c
Vitamin B-12	0.48 c	0.45 c	0.41 c	0.31 b	0.26 b	0.23 a
Folate	0.37 c	0.53 c	0.57 c	0.40 c	0.47 c	0.52 c
Pantothenic acid	0.50 c	0.63 c	0.58 c	0.47 c	0.52 c	0.54 c
Vitamin C	0.31 b	0.37 c	0.38 c	0.46 c	0.54 c	0.61 c
Sodium	0.51 c	0.60 c	0.58 c	0.22 a	0.31 c	0.47 c
Potassium	0.44 c	0.60 c	0.63 c	0.42 c	0.57 c	0.63 c
Calcium	0.41 c	0.54 c	0.55 c	0.21 a	0.33 c	0.38 c
Magnesium	0.40 c	0.56 c	0.60 c	0.40 c	0.46 c	0.55 c
Phosphorus	0.37 c	0.52 c	0.55 c	0.29 b	0.35 c	0.46 c
Iron	0.44 c	0.61 c	0.65 c	0.36 c	0.52 c	0.64 c
Zinc	0.33 c	0.43 c	0.45 c	0.33 c	0.42 c	0.53 c
Copper	0.37 c	0.58 c	0.64 c	0.40 c	0.53 c	0.56 c
Manganese	0.55 c	0.51 c	0.58 c	0.47 c	0.49 c	0.53 c

Abbreviations: SFA, saturated fatty acids; MUFA, monounsaturated fatty acids; PUFA, polyunsaturated fatty acids; EPA, eicosapentaenoic acid; DPA, Docosapentaenoic acid; DHA, Docosahexaenoic acid. ^1^ Values are expressed as Pearson correlation coefficients: a, *p* < 0.05; b, *p* < 0.01; c, *p* < 0.001. ^2^ Sum of EPA, n-3 DPA and DHA. ^3^ Sum of β-carotene, α-carotene/2 and cryptoxanthin/2. ^4^ Sum of retinol, β-carotene/12, α-carotene/24 and cryptoxanthin/24.

## Data Availability

The datasets generated and analyzed during the present study are not publicly available because of privacy and ethical restrictions imposed by the Ethics Committee of the University of Tokyo, Faculty of Medicine, but are available from the corresponding author upon reasonable request. The web and paper versions of the MDHQ used in this study are available from the corresponding author upon request.

## Supplementary Materials

The following supporting information can be downloaded at: https://www.mdpi.com/article/10.3390/nu14204270/s1, Table S1: Mean estimates of crude intakes of nutrients derived from the 4-day weighed dietary record (DR) and those derived from the web version of the Meal-based Diet History Questionnaire (MDHQ) in 111 Japanese women and 111 Japanese men, Table S2: Mean estimates of crude and energy-adjusted intakes of nutrients derived from the paper version of the Meal-based Diet History Questionnaire (MDHQ) in 111 Japanese women, Table S3: Mean estimates of crude and energy-adjusted intakes of nutrients derived from the paper version of the Meal-based Diet History Questionnaire (MDHQ) in 111 Japanese men, Table S4: Pearson correlation coefficients between crude and energy-adjusted estimates of daily intakes of nutrients derived from the 4-day weighed dietary record and those derived from the paper version of the Meal-based Diet History Questionnaire in 111 Japanese women and 111 Japanese men, Figure S1: Bland–Altman plots assessing the agreement between estimates of energy-adjusted intakes of protein (a, b), fat (c, d), and carbohydrate (e, f) derived from the 4-day weighed dietary record (DR) and those derived from the paper version of the Meal-based Diet History Questionnaire (MDHQ) in 111 Japanese women and 111 Japanese men.
