# Supplementary material for: Relative Validity of the Meal-Based Diet History Questionnaire for Estimating Nutrient Intake among Japanese Women and Men aged 30–76 Years
Source: Nutrients. 2022 Oct 13;14(20):4270. doi: 10.3390/nu14204270 (PMC9609373; doi:10.3390/nu14204270)
Supplement: Supplementary file 1 [file nutrients-14-04270-s001.zip › nutrients-1882756-supplementary.pdf]

Table S1. Mean estimates of crude intakes of nutrients derived from the 4-day weighed dietary record (DR) and those derived from the web version of the Meal-based Diet History Questionnaire (MDHQ) in 111 Japanese women and 111 Japanese men[1].

	Women			Men		
	**Unit**	**DR**	**Web MDHQ**	**Unit**	**DR**	**Web MDHQ**
Protein	g/day	64.0 ± 15.3	48.9 ± 11.9 c	g/day	79.0 ± 17.5	57.9 ± 14.8 c
Fat	g/day	60.2 ± 16.1	48.5 ± 14.2 c	g/day	72.9 ± 19.3	53.1 ± 17.2 c
SFA	g/day	18.0 ± 5.2	14.9 ± 5.2 c	g/day	20.9 ± 5.9	15.7 ± 6.8 c
MUFA	g/day	22.9 ± 7.1	17.6 ± 5.3 c	g/day	28.5 ± 8.4	19.8 ± 6.1 c
PUFA	g/day	12.2 ± 4.1	10.7 ± 3.2 c	g/day	14.9 ± 4.8	11.8 ± 3.5 c
n-6 PUFA	g/day	10.1 ± 3.4	9.0 ± 2.6 b	g/day	12.2 ± 4.1	9.8 ± 2.9 c
n-3 PUFA	g/day	2.13 ± 0.98	1.74 ± 0.62 c	g/day	2.71 ± 1.23	1.98 ± 0.66 c
Marine-origin n-3 PUFA[2]	g/day	0.66 ± 0.61	0.41 ± 0.27 c	g/day	0.87 ± 0.66	0.55 ± 0.28 c
EPA	g/day	0.21 ± 0.23	0.13 ± 0.09 c	g/day	0.28 ± 0.24	0.17 ± 0.10 c
n-3 DPA	g/day	0.07 ± 0.06	0.04 ± 0.02 c	g/day	0.09 ± 0.07	0.06 ± 0.03 c
DHA	g/day	0.38 ± 0.34	0.24 ± 0.15 c	g/day	0.50 ± 0.37	0.32 ± 0.16 c
α-linolenic acid	g/day	1.38 ± 0.69	1.28 ± 0.42	g/day	1.73 ± 0.96	1.36 ± 0.46 c
Cholesterol	mg/day	302 ± 106	228 ± 84 c	mg/day	378 ± 127	257 ± 100 c
Carbohydrate	g/day	217.2 ± 50.0	193.1 ± 52.0 c	g/day	282.1 ± 63.0	252.9 ± 66.1 c
Added sugars	g/day	30.3 ± 17.0	31.0 ± 19.9	g/day	35.5 ± 27.7	33.4 ± 25.2
Soluble dietary fiber	g/day	2.94 ± 1.03	2.16 ± 0.68 c	g/day	3.24 ± 1.02	2.34 ± 0.85 c
Insoluble dietary fiber	g/day	8.93 ± 3.50	6.98 ± 2.11 c	g/day	9.73 ± 3.11	7.72 ± 2.42 c
Total dietary fiber	g/day	12.3 ± 4.5	9.8 ± 3.1 c	g/day	13.4 ± 4.1	10.7 ± 3.5 c
Alcohol	g/day	5.67 ± 14.22	7.37 ± 16.71	g/day	21.75 ± 32.89	25.33 ± 35.31
Water	g/day	2214 ± 622	2329 ± 638	g/day	2683 ± 782	2642 ± 929
Retinol	μg/day	188 ± 347	133 ± 59	μg/day	247 ± 571	132 ± 82 a
α-carotene	μg/day	489 ± 460	512 ± 360	μg/day	515 ± 479	573 ± 458
β-carotene	μg/day	2322 ± 1530	2266 ± 1265	μg/day	2437 ± 1487	2265 ± 1378
Cryptoxanthin	μg/day	92.5 ± 185.4	124.8 ± 191.4	μg/day	66.6 ± 107.0	111.9 ± 170.0 a
β-carotene equivalent[3]	μg/day	2648 ± 1744	2620 ± 1427	μg/day	2770 ± 1701	2644 ± 1600
Retinol equivalent[4]	μg/day	409 ± 385	353 ± 141	μg/day	479 ± 598	354 ± 171 a
Vitamin D	μg/day	5.91 ± 4.43	4.23 ± 2.27 c	μg/day	7.04 ± 4.81	5.29 ± 2.39 c
α-tocopherol	mg/day	6.89 ± 2.02	5.73 ± 1.90 c	mg/day	8.22 ± 2.60	6.20 ± 2.03 c
Vitamin K	μg/day	195.2 ± 102.4	162.5 ± 74.9 c	μg/day	208.6 ± 106.3	155.7 ± 83.6 c
Thiamin	mg/day	0.86 ± 0.30	0.70 ± 0.20 c	mg/day	1.05 ± 0.29	0.81 ± 0.23 c
Riboflavin	mg/day	1.12 ± 0.32	0.95 ± 0.26 c	mg/day	1.31 ± 0.37	1.03 ± 0.34 c
Niacin	mg/day	16.2 ± 5.1	13.3 ± 3.7 c	mg/day	20.7 ± 5.8	16.7 ± 4.8 c
Vitamin B-6	mg/day	1.13 ± 0.40	0.87 ± 0.26 c	mg/day	1.38 ± 0.41	1.05 ± 0.35 c
Vitamin B-12	μg/day	4.88 ± 3.32	3.38 ± 1.84 c	μg/day	5.93 ± 3.39	4.24 ± 1.88 c
Folate	μg/day	270 ± 100	220 ± 72 c	μg/day	304 ± 115	237 ± 83 c
Pantothenic acid	mg/day	5.18 ± 1.24	4.20 ± 1.06 c	mg/day	6.20 ± 1.58	4.81 ± 1.31 c
Vitamin C	mg/day	79 ± 35	68 ± 28 b	mg/day	90 ± 39	70 ± 32 c
Sodium	mg/day	3340 ± 1018	3221 ± 970	mg/day	4335 ± 1472	3931 ± 1163 a
Potassium	mg/day	2242 ± 656	1968 ± 542 c	mg/day	2536 ± 686	2146 ± 629 c
Calcium	mg/day	477 ± 195	415 ± 135 c	mg/day	505 ± 210	399 ± 178 c
Magnesium	mg/day	244 ± 75	219 ± 60 c	mg/day	281 ± 85	247 ± 73 c
Phosphorus	mg/day	951 ± 241	774 ± 198 c	mg/day	1127 ± 285	883 ± 276 c
Iron	mg/day	6.72 ± 2.17	5.41 ± 1.59 c	mg/day	7.70 ± 2.07	5.96 ± 1.75 c
Zinc	mg/day	7.56 ± 1.90	6.10 ± 1.40 c	mg/day	9.20 ± 2.14	7.23 ± 1.90 c
Copper	mg/day	1.00 ± 0.31	0.84 ± 0.23 c	mg/day	1.18 ± 0.31	0.99 ± 0.26 c
Manganese	mg/day	2.86 ± 0.98	2.85 ± 1.13	mg/day	3.56 ± 1.28	3.42 ± 1.39

Abbreviations: SFA, saturated fatty acids; MUFA, monounsaturated fatty acids; PUFA, polyunsaturated fatty acids; EPA, eicosapentaenoic acid; DPA, Docosapentaenoic acid; DHA, Docosahexaenoic acid. [1] Values are means ± standard deviations. The values derived from the MDHQ were compared with those derived from the DR using the paired *t*-test: a, p <0.05; b, p <0.01; c, p <0.001. [2] Sum of EPA, n-3 DPA, and DHA. [3] Sum of β-carotene, α-carotene/2, and cryptoxanthin/2. [4] Sum of retinol, β-carotene/12, α-carotene/24, and cryptoxanthin/24.

Table S2. Mean estimates of crude and energy-adjusted intakes of nutrients derived from the paper version of the Meal-based Diet History Questionnaire (MDHQ) in 111 Japanese women[1].

	**Crude model**		**Residual model**		**Density model**	
	**Unit**	**Paper MDHQ**	**Unit**	**Paper MDHQ**	**Unit**	**Paper MDHQ**
Protein	g/day	50.8 ± 12.4 c	g/day	50.8 ± 7.4 c	% of energy	13.5 ± 2.0 c
Fat	g/day	50.0 ± 14.5 c	g/day	50.0 ± 8.6 c	% of energy	29.7 ± 5.3 b
SFA	g/day	15.3 ± 5.1 c	g/day	15.3 ± 3.4 c	% of energy	9.1 ± 2.0
MUFA	g/day	18.1 ± 5.3 c	g/day	18.1 ± 3.3 c	% of energy	10.8 ± 2.0 c
PUFA	g/day	11.1 ± 3.3 b	g/day	11.1 ± 2.3 c	% of energy	6.6 ± 1.4
n-6 PUFA	g/day	9.3 ± 2.7 a	g/day	9.3 ± 1.9 c	% of energy	5.5 ± 1.1 c
n-3 PUFA	g/day	1.80 ± 0.67 c	g/day	1.80 ± 0.52 c	% of energy	1.06 ± 0.31 c
Marine-origin n-3 PUFA[2]	g/day	0.43 ± 0.27 c	g/day	0.43 ± 0.24 c	% of energy	0.25 ± 0.14
EPA	g/day	0.13 ± 0.09 c	g/day	0.13 ± 0.08 c	% of energy	0.08 ± 0.05
n-3 DPA	g/day	0.04 ± 0.02 c	g/day	0.04 ± 0.02 c	% of energy	0.03 ± 0.01
DHA	g/day	0.25 ± 0.15 c	g/day	0.25 ± 0.14 c	% of energy	0.15 ± 0.08
α-linolenic acid	g/day	1.31 ± 0.44	g/day	1.31 ± 0.34	% of energy	0.78 ± 0.20 c
Cholesterol	mg/day	243 ± 95 c	mg/day	243 ± 70 c	mg/1000 kcal	159 ± 47 b
Carbohydrate	g/day	198.9 ± 48.5 c	g/day	198.9 ± 28.1 c	% of energy	52.9 ± 7.3 c
Added sugars	g/day	31.6 ± 18.3	g/day	31.6 ± 15.6	% of energy	8.2 ± 4.1 b
Soluble dietary fiber	g/day	2.31 ± 0.72 c	g/day	2.31 ± 0.49 c	g/1000 kcal	1.52 ± 0.34 c
Insoluble dietary fiber	g/day	7.35 ± 2.26 c	g/day	7.35 ± 1.47 c	g/1000 kcal	4.85 ± 0.98 b
Total dietary fiber	g/day	10.4 ± 3.3 c	g/day	10.4 ± 2.2 c	g/1000 kcal	6.9 ± 1.5
Alcohol	g/day	6.80 ± 16.13	g/day	6.80 ± 15.94	% of energy	3.04 ± 6.87 a
Water	g/day	2405 ± 651 b	g/day	2405 ± 535 b	g/1000 kcal	1616 ± 385 c
Retinol	μg/day	137 ± 63	μg/day	137 ± 50	μg/1000 kcal	90 ± 33
α-carotene	μg/day	612 ± 415 b	μg/day	612 ± 394 b	μg/1000 kcal	403 ± 269 c
β-carotene	μg/day	2484 ± 1399	μg/day	2484 ± 1267	μg/1000 kcal	1633 ± 869 c
Cryptoxanthin	μg/day	149.9 ± 199.3 b	μg/day	149.9 ± 193.4 c	μg/1000 kcal	96.1 ± 123.8 c
β-carotene equivalent[3]	μg/day	2897 ± 1615	μg/day	2897 ± 1459	μg/1000 kcal	1904 ± 999 c
Retinol equivalent[4]	μg/day	380 ± 166	μg/day	380 ± 135	μg/1000 kcal	250 ± 94
Vitamin D	μg/day	4.66 ± 2.69 b	μg/day	4.66 ± 2.26 c	μg/1000 kcal	3.03 ± 1.44 a
α-tocopherol	mg/day	5.95 ± 1.90 c	mg/day	5.95 ± 1.29 c	mg/1000 kcal	3.92 ± 0.90
Vitamin K	μg/day	169.1 ± 76.5 b	μg/day	169.1 ± 67.5 c	μg/1000 kcal	112.1 ± 45.7
Thiamin	mg/day	0.73 ± 0.20 c	mg/day	0.73 ± 0.14 c	mg/1000 kcal	0.48 ± 0.09
Riboflavin	mg/day	1.00 ± 0.30 c	mg/day	1.00 ± 0.21 c	mg/1000 kcal	0.67 ± 0.14
Niacin	mg/day	13.5 ± 4.0 c	mg/day	13.5 ± 2.9 c	mg/1000 kcal	9.0 ± 1.9 a
Vitamin B-6	mg/day	0.89 ± 0.29 c	mg/day	0.89 ± 0.20 c	mg/1000 kcal	0.59 ± 0.13 c
Vitamin B-12	μg/day	3.59 ± 1.95 c	μg/day	3.59 ± 1.70 c	μg/1000 kcal	2.35 ± 1.10 b
Folate	μg/day	225 ± 78 c	μg/day	225 ± 57 c	μg/1000 kcal	149 ± 41 a
Pantothenic acid	mg/day	4.37 ± 1.18 c	mg/day	4.37 ± 0.67 c	mg/1000 kcal	2.89 ± 0.45 b
Vitamin C	mg/day	68 ± 30 b	mg/day	68 ± 24 c	mg/1000 kcal	45 ± 17
Sodium	mg/day	3380 ± 996	mg/day	3380 ± 683	mg/1000 kcal	2235 ± 439 c
Potassium	mg/day	2040 ± 613 b	mg/day	2040 ± 411 c	mg/1000 kcal	1350 ± 279
Calcium	mg/day	446 ± 157	mg/day	446 ± 120 a	mg/1000 kcal	296 ± 82 a
Magnesium	mg/day	226 ± 67 a	mg/day	226 ± 45 c	mg/1000 kcal	150 ± 31 c
Phosphorus	mg/day	807 ± 223 c	mg/day	807 ± 136 c	mg/1000 kcal	534 ± 91 a
Iron	mg/day	5.61 ± 1.77 c	mg/day	5.61 ± 1.13 c	mg/1000 kcal	3.70 ± 0.77 b
Zinc	mg/day	6.28 ± 1.48 c	mg/day	6.28 ± 0.84 c	mg/1000 kcal	4.18 ± 0.56 b
Copper	mg/day	0.87 ± 0.24 c	mg/day	0.87 ± 0.14 c	mg/1000 kcal	0.57 ± 0.09
Manganese	mg/day	2.86 ± 1.19	mg/day	2.86 ± 1.19	mg/1000 kcal	1.92 ± 0.74 c

Abbreviations: SFA, saturated fatty acids; MUFA, monounsaturated fatty acids; PUFA, polyunsaturated fatty acids; EPA, eicosapentaenoic acid; DPA, Docosapentaenoic acid; DHA, Docosahexaenoic acid. [1] Values are means ± standard deviations. The values derived from the MDHQ were compared with those derived from the 4-day weighed dietary record (shown in Table S1 for crude estimates and Table 2 for energy-adjusted estimates by the residual model and by the density model) using the paired *t*-test: a, p <0.05; b, p <0.01; c, p <0.001. [2] Sum of EPA, n-3 DPA, and DHA. [3] Sum of β-carotene, α-carotene/2, and cryptoxanthin/2. [4] Sum of retinol, β-carotene/12, α-carotene/24, and cryptoxanthin/24.

Table S3. Mean estimates of crude and energy-adjusted intakes of nutrients derived from the paper version of the Meal-based Diet History Questionnaire (MDHQ) in 111 Japanese men[1].

	Crude model		Residual model		Density model	
	**Unit**	**Paper MDHQ**	**Unit**	**Paper MDHQ**	**Unit**	**Paper MDHQ**
Protein	g/day	58.5 ± 14.4 c	g/day	58.5 ± 8.1 c	% of energy	12.4 ± 1.8 c
Fat	g/day	52.6 ± 14.4 c	g/day	52.6 ± 9.5 c	% of energy	25.1 ± 4.8 c
SFA	g/day	15.4 ± 4.8 c	g/day	15.4 ± 3.4 c	% of energy	7.3 ± 1.6 c
MUFA	g/day	19.6 ± 5.3 c	g/day	19.6 ± 3.6 c	% of energy	9.4 ± 1.9 c
PUFA	g/day	11.8 ± 3.5 c	g/day	11.8 ± 2.6 c	% of energy	5.6 ± 1.4
n-6 PUFA	g/day	9.8 ± 2.9 c	g/day	9.8 ± 2.1 c	% of energy	4.7 ± 1.1 c
n-3 PUFA	g/day	1.96 ± 0.69 c	g/day	1.96 ± 0.54 c	% of energy	0.93 ± 0.28 a
Marine-origin n-3 PUFA[2]	g/day	0.54 ± 0.30 c	g/day	0.54 ± 0.26 c	% of energy	0.26 ± 0.12
EPA	g/day	0.17 ± 0.10 c	g/day	0.17 ± 0.09 c	% of energy	0.08 ± 0.04
n-3 DPA	g/day	0.06 ± 0.03 c	g/day	0.06 ± 0.02 c	% of energy	0.03 ± 0.01
DHA	g/day	0.32 ± 0.17 c	g/day	0.32 ± 0.15 c	% of energy	0.15 ± 0.07
α-linolenic acid	g/day	1.35 ± 0.46 c	g/day	1.35 ± 0.37 c	% of energy	0.65 ± 0.19 c
Cholesterol	mg/day	273 ± 112 c	mg/day	273 ± 84 c	mg/1000 kcal	142 ± 45 c
Carbohydrate	g/day	248.5 ± 61.1 c	g/day	248.5 ± 37.9 c	% of energy	52.8 ± 8.2 c
Added sugars	g/day	31.7 ± 22.5	g/day	31.7 ± 20.8	% of energy	6.6 ± 4.1
Soluble dietary fiber	g/day	2.37 ± 0.83 c	g/day	2.37 ± 0.67 c	g/1000 kcal	1.26 ± 0.38 c
Insoluble dietary fiber	g/day	7.82 ± 2.53 c	g/day	7.82 ± 1.96 c	g/1000 kcal	4.14 ± 1.06 a
Total dietary fiber	g/day	10.9 ± 3.7 c	g/day	10.9 ± 2.9 c	g/1000 kcal	5.7 ± 1.6
Alcohol	g/day	23.77 ± 29.47	g/day	23.77 ± 27.31	% of energy	8.30 ± 9.98 c
Water	g/day	2659 ± 778	g/day	2659 ± 614	g/1000 kcal	1417 ± 324 c
Retinol	μg/day	132 ± 69 a	μg/day	132 ± 60 a	μg/1000 kcal	69 ± 33
α-carotene	μg/day	593 ± 469	μg/day	593 ± 439	μg/1000 kcal	307 ± 234 c
β-carotene	μg/day	2427 ± 1508	μg/day	2427 ± 1382	μg/1000 kcal	1267 ± 747 b
Cryptoxanthin	μg/day	127.9 ± 155.6 c	μg/day	127.9 ± 154.8 c	μg/1000 kcal	67.9 ± 85.0 c
β-carotene equivalent[3]	μg/day	2821 ± 1743	μg/day	2821 ± 1597	μg/1000 kcal	1472 ± 862 b
Retinol equivalent[4]	μg/day	370 ± 185 a	μg/day	370 ± 161 a	μg/1000 kcal	193 ± 88
Vitamin D	μg/day	5.22 ± 2.39 c	μg/day	5.22 ± 2.00 c	μg/1000 kcal	2.75 ± 1.09
α-tocopherol	mg/day	6.19 ± 1.98 c	mg/day	6.19 ± 1.52 c	mg/1000 kcal	3.27 ± 0.84 c
Vitamin K	μg/day	172.0 ± 104.9 c	μg/day	172.0 ± 99.7 c	μg/1000 kcal	91.8 ± 64.3
Thiamin	mg/day	0.82 ± 0.21 c	mg/day	0.82 ± 0.16 c	mg/1000 kcal	0.44 ± 0.09 a
Riboflavin	mg/day	1.06 ± 0.33 c	mg/day	1.06 ± 0.25 c	mg/1000 kcal	0.56 ± 0.14
Niacin	mg/day	16.8 ± 5.0 c	mg/day	16.8 ± 3.6 c	mg/1000 kcal	8.9 ± 2.1
Vitamin B-6	mg/day	1.05 ± 0.33 c	mg/day	1.05 ± 0.23 c	mg/1000 kcal	0.55 ± 0.13 c
Vitamin B-12	μg/day	4.33 ± 2.26 c	μg/day	4.33 ± 1.88 c	μg/1000 kcal	2.25 ± 0.96 a
Folate	μg/day	245 ± 89 c	μg/day	245 ± 76 c	μg/1000 kcal	130 ± 43
Pantothenic acid	mg/day	4.88 ± 1.37 c	mg/day	4.88 ± 0.87 c	mg/1000 kcal	2.58 ± 0.50 c
Vitamin C	mg/day	72 ± 33 c	mg/day	72 ± 30 c	mg/1000 kcal	38 ± 17
Sodium	mg/day	3973 ± 1143 b	mg/day	3973 ± 849 c	mg/1000 kcal	2106 ± 456 c
Potassium	mg/day	2197 ± 681 c	mg/day	2197 ± 495 c	mg/1000 kcal	1161 ± 276 a
Calcium	mg/day	414 ± 157 c	mg/day	414 ± 119 c	mg/1000 kcal	217 ± 63
Magnesium	mg/day	252 ± 76 c	mg/day	252 ± 51 c	mg/1000 kcal	133 ± 28 c
Phosphorus	mg/day	894 ± 251 c	mg/day	894 ± 147 c	mg/1000 kcal	471 ± 80 b
Iron	mg/day	6.15 ± 1.89 c	mg/day	6.15 ± 1.38 c	mg/1000 kcal	3.25 ± 0.82 a
Zinc	mg/day	7.39 ± 1.81 c	mg/day	7.39 ± 1.10 c	mg/1000 kcal	3.92 ± 0.60 a
Copper	mg/day	1.00 ± 0.27 c	mg/day	1.00 ± 0.17 c	mg/1000 kcal	0.53 ± 0.10
Manganese	mg/day	3.44 ± 1.20	mg/day	3.44 ± 1.20	mg/1000 kcal	1.85 ± 0.65 c

Abbreviations: SFA, saturated fatty acids; MUFA, monounsaturated fatty acids; PUFA, polyunsaturated fatty acids; EPA, eicosapentaenoic acid; DPA, Docosapentaenoic acid; DHA, Docosahexaenoic acid. [1] Values are means ± standard deviations. The values derived from the MDHQ were compared with those derived from the 4-day weighed dietary record (shown in Table S1 for crude estimates and Table 2 for energy-adjusted estimates by the residual model and by the density model) using the paired *t*-test: a, $p < 0.05$; b, $p < 0.01$; c, $p < 0.001$. [2] Sum of EPA, n-3 DPA, and DHA. [3] Sum of β-carotene, α-carotene/2, and cryptoxanthin/2. [4] Sum of retinol, β-carotene/12, α-carotene/24, and cryptoxanthin/24.

Table S4. Pearson correlation coefficients between crude and energy-adjusted estimates of daily intakes of nutrients derived from the 4-day weighed dietary record and those derived from the paper version of the Meal-based Diet History Questionnaire in 111 Japanese women and 111 Japanese men[1].

	Women			Men		
	**Crude model**	**Residual model**	**Density model**	**Crude model**	**Residual model**	**Density model**
Protein	0.40 c	0.59 c	0.56 c	0.46 c	0.45 c	0.48 c
Fat	0.31 c	0.51 c	0.42 c	0.23 a	0.40 c	0.45 c
SFA	0.29 b	0.43 c	0.40 c	0.24 a	0.49 c	0.50 c
MUFA	0.29 b	0.44 c	0.35 c	0.18	0.29 b	0.35 c
PUFA	0.38 c	0.50 c	0.42 c	0.32 c	0.37 c	0.41 c
n-6 PUFA	0.38 c	0.48 c	0.41 c	0.31 b	0.36 c	0.41 c
n-3 PUFA	0.33 c	0.42 c	0.34 c	0.29 b	0.28 b	0.19 a
Marine-origin n-3 PUFA[2]	0.55 c	0.61 c	0.55 c	0.41 c	0.35 c	0.28 b
EPA	0.55 c	0.61 c	0.56 c	0.40 c	0.36 c	0.29 b
n-3 DPA	0.49 c	0.54 c	0.45 c	0.36 c	0.32 c	0.28 b
DHA	0.55 c	0.61 c	0.54 c	0.42 c	0.34 c	0.26 b
α-linolenic acid	0.05	0.03	0.01	0.09	0.13	0.10
Cholesterol	0.46 c	0.46 c	0.38 c	0.56 c	0.49 c	0.46 c
Carbohydrate	0.53 c	0.70 c	0.65 c	0.47 c	0.71 c	0.76 c
Added sugars	0.32 c	0.38 c	0.41 c	0.20 a	0.33 c	0.41 c
Soluble dietary fiber	0.45 c	0.58 c	0.58 c	0.42 c	0.51 c	0.56 c
Insoluble dietary fiber	0.40 c	0.55 c	0.60 c	0.54 c	0.65 c	0.67 c
Total dietary fiber	0.44 c	0.62 c	0.65 c	0.54 c	0.67 c	0.68 c
Alcohol	0.76 c	0.77 c	0.83 c	0.86 c	0.75 c	0.87 c
Water	0.31 c	0.21 a	0.21 a	0.39 c	0.29 b	0.29 b
Retinol	0.20 a	0.15	0.15	0.35 c	0.37 c	0.27 b
α-carotene	0.50 c	0.56 c	0.57 c	0.42 c	0.40 c	0.44 c
β-carotene	0.47 c	0.57 c	0.57 c	0.42 c	0.42 c	0.45 c
Cryptoxanthin	0.56 c	0.56 c	0.51 c	0.41 c	0.41 c	0.35 c
β-carotene equivalent[3]	0.48 c	0.59 c	0.59 c	0.41 c	0.41 c	0.44 c
Retinol equivalent[4]	0.26 b	0.25 b	0.26 b	0.26 b	0.25 b	0.20 a
Vitamin D	0.42 c	0.47 c	0.42 c	0.36 c	0.29 b	0.25 b
α-tocopherol	0.42 c	0.50 c	0.45 c	0.31 b	0.41 c	0.44 c
Vitamin K	0.53 c	0.59 c	0.57 c	0.44 c	0.47 c	0.52 c
Thiamin	0.35 c	0.42 c	0.46 c	0.23 a	0.31 b	0.38 c
Riboflavin	0.47 c	0.52 c	0.50 c	0.53 c	0.60 c	0.65 c
Niacin	0.37 c	0.43 c	0.43 c	0.45 c	0.28 b	0.31 c
Vitamin B-6	0.45 c	0.58 c	0.61 c	0.57 c	0.48 c	0.52 c
Vitamin B-12	0.51 c	0.50 c	0.42 c	0.45 c	0.33 c	0.26 b
Folate	0.49 c	0.57 c	0.57 c	0.45 c	0.46 c	0.49 c
Pantothenic acid	0.48 c	0.56 c	0.54 c	0.59 c	0.63 c	0.64 c
Vitamin C	0.41 c	0.46 c	0.50 c	0.43 c	0.50 c	0.54 c
Sodium	0.47 c	0.61 c	0.55 c	0.44 c	0.43 c	0.43 c
Potassium	0.48 c	0.63 c	0.64 c	0.51 c	0.62 c	0.68 c
Calcium	0.5 c	0.59 c	0.59 c	0.41 c	0.48 c	0.50 c
Magnesium	0.45 c	0.66 c	0.66 c	0.51 c	0.59 c	0.63 c
Phosphorus	0.43 c	0.60 c	0.59 c	0.45 c	0.51 c	0.54 c
Iron	0.49 c	0.64 c	0.61 c	0.53 c	0.59 c	0.63 c
Zinc	0.42 c	0.55 c	0.48 c	0.48 c	0.56 c	0.57 c
Copper	0.44 c	0.61 c	0.63 c	0.53 c	0.62 c	0.61 c
Manganese	0.65 c	0.63 c	0.64 c	0.56 c	0.59 c	0.69 c

Abbreviations: SFA, saturated fatty acids; MUFA, monounsaturated fatty acids; PUFA, polyunsaturated fatty acids; EPA, eicosapentaenoic acid; DPA, Docosapentaenoic acid; DHA, Docosahexaenoic acid. [1] Values are expressed as Pearson correlation coefficients: a, $p < 0.05$; b, $p < 0.01$; c, $p < 0.001$. [2] Sum of EPA, n-3 DPA, and DHA. [3] Sum of β-carotene, α-carotene/2, and cryptoxanthin/2. [4] Sum of retinol, β-carotene/12, α-carotene/24, and cryptoxanthin/24.

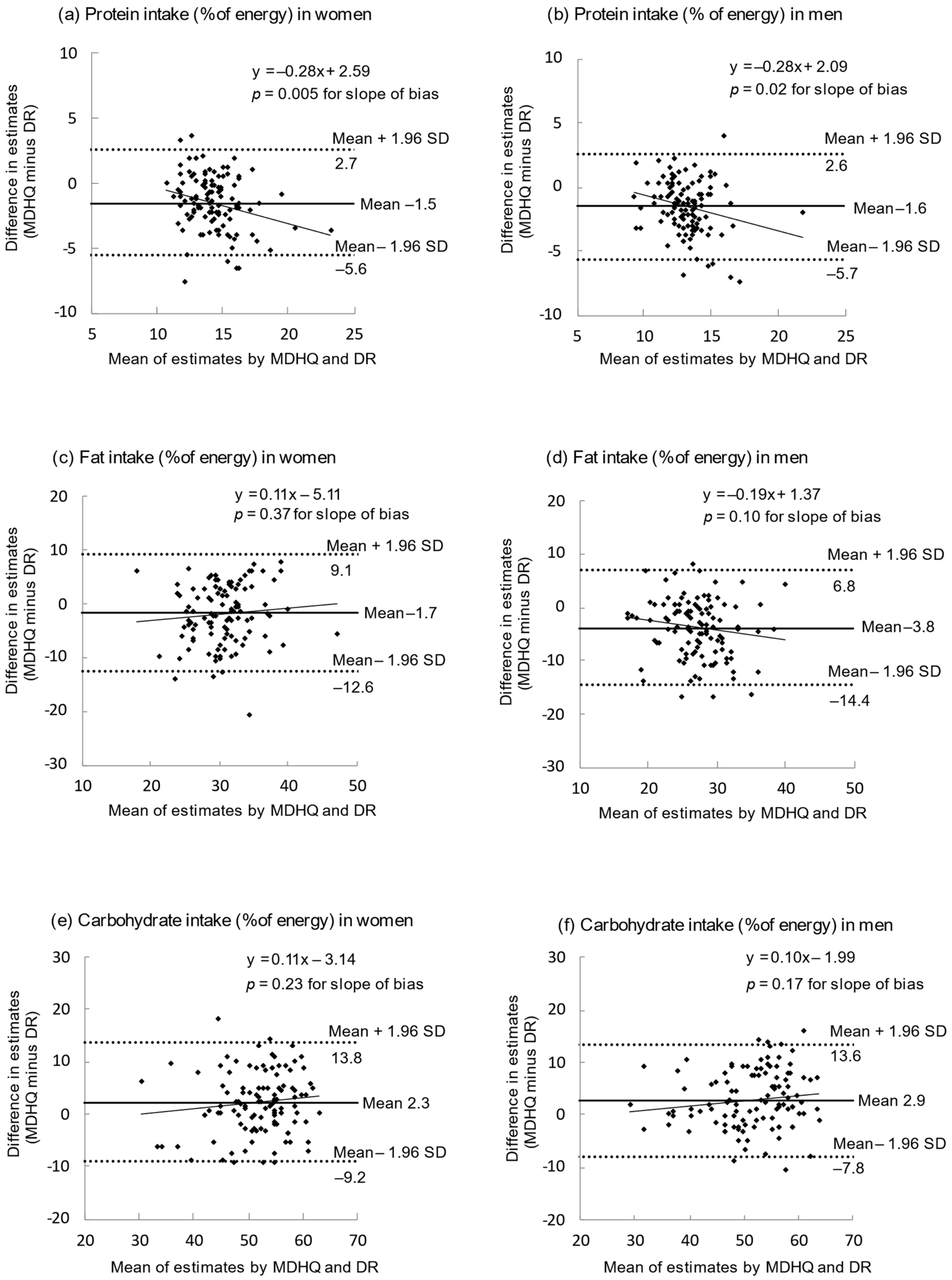

Figure S1. Bland–Altman plots assessing the agreement between estimates of energy-adjusted intakes of protein (**a, b**), fat (**c, d**), and carbohydrate (**e, f**) derived from the 4-day weighed dietary record (DR) and those derived from the paper version of the Meal-based Diet History Questionnaire (MDHQ) in 111 Japanese women and 111 Japanese men. Energy adjustment was done using the density model. SD, standard deviation.
